# Relationship of Perivascular Space Markers With Incident Dementia in Cerebral Small Vessel Disease

**DOI:** 10.1161/STROKEAHA.123.045857

**Published:** 2024-03-11

**Authors:** Hui Hong, Daniel J. Tozer, Hugh S. Markus

**Affiliations:** Department of Clinical Neurosciences, University of Cambridge, United Kingdom (H.H., D.J.T., H.S.M.).; Department of Radiology, Second Affiliated Hospital of Zhejiang University, School of Medicine, Hangzhou, China (H.H.).

**Keywords:** cerebral small vessel disease, cognitive dysfunction, dementia, white matter

## Abstract

**BACKGROUND:**

Recent studies, using diffusion tensor image analysis along the perivascular space (DTI-ALPS), suggest impaired perivascular space (PVS) function in cerebral small vessel disease, but they were cross-sectional, making inferences on causality difficult. We determined associations between impaired PVS, measured using DTI-ALPS and PVS volume, and cognition and incident dementia.

**METHODS:**

In patients with lacunar stroke and confluent white matter hyperintensities, without dementia at baseline, recruited prospectively in a single center, magnetic resonance imaging was performed annually for 3 years, and cognitive assessments, including global, memory, executive function, and processing speed, were performed annually for 5 years. We determined associations between DTI-ALPS and PVS volume with cerebral small vessel disease imaging markers (white matter hyperintensity volume, lacunes, and microbleeds) at baseline and with changes in imaging markers. We determined whether DTI-ALPS and PVS volume at baseline and change over 3 years predicted incident dementia. Analyses were controlled for conventional diffusion tensor image metrics using 2 markers (median mean diffusivity [MD] and peak width of skeletonized MD) and adjusted for age, sex, and vascular risk factors.

**RESULTS:**

A total of 120 patients, mean age 70.0 years and 65.0% male, were included. DTI-ALPS declined over 3 years, while no change in PVS volume was found. Neither DTI-ALPS nor PVS volume was associated with cerebral small vessel disease imaging marker progression. Baseline DTI-ALPS was associated with changes in global cognition (β=0.142, *P*=0.032), executive function (β=0.287, *P*=0.027), and long-term memory (β=0.228, *P*=0.027). Higher DTI-ALPS at baseline predicted a lower risk of dementia (hazard ratio, 0.328 [0.183–0.588]; *P*<0.001), and this remained significant after including median MD as a covariate (hazard ratio, 0.290 [0.139–0.602]; *P*<0.001). Change in DTI-ALPS predicted dementia conversion (hazard ratio, 0.630 [0.428–0.964]; *P*=0.048), but when peak width of skeletonized MD and median MD were entered as covariates, the association was not significant. There was no association between baseline PVS volume, or PVS change over 3 years, and conversion to dementia.

**CONCLUSIONS:**

DTI-ALPS predicts future dementia risk in patients with lacunar strokes and confluent white matter hyperintensities. However, the weakening of the association between change in DTI-ALPS and incident dementia after controlling for peak width of skeletonized MD and median MD suggests part of the signal may represent conventional diffusion tensor image metrics. PVS volume is not a predictor of future dementia risk.

Cerebral small vessel disease (CSVD) describes disease in the small arteries, arterioles, venules, and capillaries in the brain.^1^ It causes a quarter of strokes, is the most common pathology underlying vascular dementia,^1^ and its presence increases the likelihood that neurodegenerative pathologies like Alzheimer disease result in clinical dementia.^2^

Perivascular space (PVS) dysfunction has recently emerged as a pathophysiological mechanism in CSVD progression.^3^ The PVS comprises a network of spaces surrounding cerebral microvessels, serving as conduits for fluid transport, exchange between cerebrospinal fluid (CSF) and interstitial fluid, and the clearance of waste products from the brain.^4^

The enlargement of PVS, leading to their visibility on magnetic resonance imaging (MRI), indicates potential obstruction by protein and cell debris, resulting in the stagnation of fluid drainage.^5^ This phenomenon is commonly observed in CSVD. Both the rating scale for PVS severity and the PVS volume quantification methods are associated with other CSVD imaging markers.^6–9^ Spatial relationship studies have revealed that white matter hyperintensities (WMH) often surround enlarged PVS, suggesting that these enlarged PVS could be a factor in the progression of WMH and possibly other CSVD imaging markers.^10,11^ However, longitudinal analyses exploring its correlation with CSVD progression are lacking. Additionally, PVS enlargement has been linked to all cause dementia,^12,13^ although not all studies have replicated this association.^14–16^

The diffusion tensor imaging analysis along the PVS (DTI-ALPS) method provides an additional marker for assessing the PVS function.^17^ It measures the directionality and magnitude of water diffusion along PVS.^18^ Previous studies using this approach have revealed correlations between DTI-ALPS and vascular risk factors,^19^ with other imaging markers of CSVD,^20^ and with cognitive performance.^21^ However, it is important to note that the existing studies have been primarily cross-sectional, and whether the associations reported are causal is uncertain. One important consideration is the potential confounding effect of conventional diffusion tensor imaging (DTI) metrics, such as mean diffusivity (MD) and peak width of skeletonized MD (PSMD), on DTI-ALPS, because the DTI-ALPS metric is obtained from the DTI sequence. MD assesses general alterations in brain tissues without directional specificity, while PSMD evaluates variations of MD on a skeleton. This is relevant because MD has demonstrated high sensitivity as a disease marker in CSVD and is associated with both cognition and the prediction of future dementia risk.^22^ Therefore, it is important to establish that any relationship between DTI-ALPS and CSVD is independent of conventional DTI metrics.

To better understand the significance of PVS markers and their relationship to cognition in CSVD, in a prospective longitudinal cohort of patients with lacunar and confluent WMH CSVD, we examined associations between PVS markers and MRI markers of CSVD severity and whether they predicted incident dementia over a 5-year follow-up. When examining associations with DTI-ALPS, we controlled for conventional DTI metrics.

## METHODS

The study was registered (www.ukctg.nihr.ac.uk; study ID: 4577) and approved by a local research ethics committee (London-Wandsworth). Participants provided written informed consent. On reasonable request, data from this study are available from the corresponding author. This study follows the Strengthening the Reporting of Observational Studies in Epidemiology reporting guideline.^23^

### Overall Study Design

We used data from the prospective SCANS study (St. George’s Cognition and Neuroimaging in Stroke). This study recruited nondemented patients with a clinical lacunar stroke associated with an anatomically appropriate lacunar infarct and confluent WMH. Full inclusion and exclusion criteria have been published previously.^24^

MRI and cognitive assessments were performed at baseline. Participants were invited back annually for repeated cognitive testing and MRI scanning for a period of 3 years. Subsequently, 2 further annual assessments of cognitive function were conducted at years 4 and 5 without MRI. A longitudinal cohort flowchart is shown in Figure S1.

### Clinical Assessments

Cerebrovascular risk factors, such as hypertension, diabetes, hypercholesterolemia, and smoking history, were documented. Hypertension was defined as either systolic blood pressure >140 mm Hg, diastolic blood pressure >90 mm Hg, or on treatment. Hypercholesterolemia was defined as a random total cholesterol of >5.2 mmol/L or on treatment. Smoking was divided into current smoker, ex-smoker, and never smoker. Diabetes was defined as being on drug or insulin treatment. Premorbid intelligence quotient was estimated using the restandardized National Adult Reading Test (NART-IQ).

### Image Acquisition

MRI scanning took place at baseline and over 3 yearly follow-up sessions on a 1.5T General Electric Signa HDxt MRI system. The imaging protocols included fluid-attenuated inversion recovery, T2, T2*-weighted gradient echo images, T1, and diffusion imaging. The T1 image was acquired using spoiled gradient echo recalled T1-weighted 3-dimensional coronal sequence: repetition time/echo time, 11.5/5 ms; field of view, 240×240 mm^2^; matrix, 256×192; flip angle, 18°; 176 coronal slices of 1.1-mm thickness reconstructed to an in-plane resolution of 1.1 mm. Diffusion images were acquired using a spin-echo planar sequence with isotropic resolution (2.5 mm^3^) and 25 diffusion gradient directions at b=1000 s/mm^2^ in positive and negative gradient directions. Eight echo planar images were acquired without a diffusion gradient (b=0 s/mm^2^). Acquisition details of other sequences have been described previously.^25^

### Neuropsychological Assessment

A detailed neuropsychological assessment was performed, including memory, executive function, and processing speed, measured using standardized tests as previously described.^24^ Age-standardized test scores were used to derive a measure of global cognition. Subdomain scores for executive function, processing speed, and long-term memory were also derived.^24^ Dementia was defined according to the Diagnostic and Statistical Manual of Mental Disorders 5 criteria^26^ and was diagnosed if individuals met one of the following criteria during follow-up:

1. A clinical diagnosis of dementia by a dementia expert.
2. On review of cognitive assessments together with medical records by a neurologist and clinical neuropsychologist, blinded to MRI and risk factor information, who agreed that the clinical picture met the Diagnostic and Statistical Manual of Mental Disorders 5 criteria.
3. A Mini-Mental State Examination score consistently <24 is indicative of cognitive impairment and reduced capabilities in daily living, as measured by a score ≤7 on the instrumental activities of daily living.

### Image Processing

#### CSVD Imaging Markers

1. Visible CSVD imaging markers: WMH volume was measured using the semiautomated tool DISPUNC as previously described.^24^ A single consultant neuroradiologist evaluated the T1-weighted and fluid-attenuated inversion recovery images for lacunar infarcts, defined as a CSF-filled cavity within the white matter (WM) or subcortical regions, between 3 and 15 mm in diameter.^24^ They were distinguished from PVS by published criteria.^15^ Microbleeds were identified on gradient echo as well-defined focal areas of low signal <10 mm in diameter, as previously described.^15^
2. DTI markers: a tensor model was used to analyze the diffusion data; from this analysis, the median MD in WM was measured as a conventional marker of DTI metrics using histogram analysis as previously described.^24^ In addition, an automated measure of PSMD was determined.^27^

### Tissue Segmentation

The tissue segmentation steps have been previously described.^28^ Briefly, a group average template was first created, and the T1-weighted and fluid-attenuated inversion recovery images were warped to this space. Second, the warped T1-weighted and fluid-attenuated inversion recovery images were used to create population-specific tissue probability maps. Third, the new tissue probability maps were used to resegment the native images, creating gray matter, WM, CSF, and WMH tissue classes. These were then combined with the manually defined lacune regions of interest, resulting in 5 tissue classes per individual. Finally, a tissue repair step was performed to generate repaired gray matter, WM, and CSF maps for each individual data set.

### DTI-ALPS and PVS Imaging Markers

**Figure 1.**
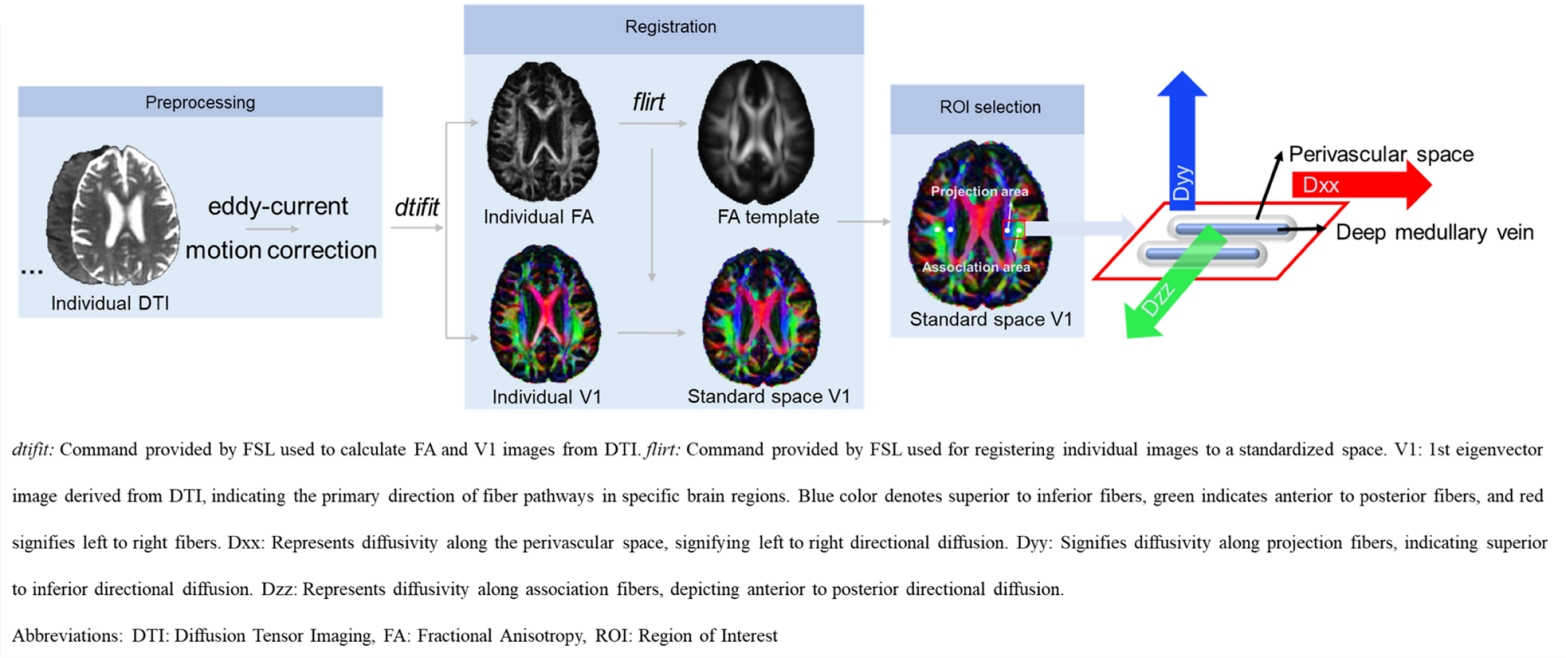
**Diffusion tensor image (DTI) analysis along the perivascular space (DTI-ALPS) workflow.** dtifit: command provided by FSL used to calculate fractional anisotropy (FA) and V1 images from DTI. flirt: command provided by FSL used for registering individual images to a standardized space. V1 is the first eigenvector image derived from DTI, indicating the primary direction of fiber pathways in specific brain regions. Blue color denotes superior to inferior fibers, green indicates anterior to posterior fibers, and red signifies left to right fibers. Dxx indicates diffusivity along the perivascular space, signifying left to right directional diffusion; Dyy, diffusivity along projection fibers, indicating superior to inferior directional diffusion; Dzz, diffusivity along association fibers, depicting anterior to posterior directional diffusion; FSL, Oxford Centre for Functional Magnetic Resonance Imaging of the Brain Software Library; and ROI, region of interest.

1. DTI-ALPS calculation: diffusion preprocessing has been described previously,^29^ including eddy current and head motion correction. DTI-ALPS was calculated according to a published method.^18^ Fractional anisotropy and diffusivity maps (Dxx, Dyy, Dzz) were acquired from preprocessed DTI using the Oxford Centre for Functional Magnetic Resonance Imaging of the Brain Software Library ([FSL]; version 6.0.3; http://www.fmrib.ox.ac.uk/fsl) dtifit–save tensor tool. Individual fractional anisotropy map was coregistered to the Montreal Neurological Institute space fractional anisotropy map template (Johns Hopkins University-International Consortium for Brain Mapping-fractional anisotropy-1mm [JHU-ICBM-FA-1mm]) by linear registration using FSL flirt tool. The acquired transformation matrix was also applied to individual (Dxx, Dyy, Dzz) maps. Four 6-mm-diameter sphere regions of interest were designed in the Montreal Neurological Institute space. The coordinate centers of regions of interest were (24, −12, 24), (−28, −12, 24), (36, −12, 24), and (−40, −12, 24), respectively. Finally, manual correction was performed to confirm the accuracy of registration and the location of regions of interest for each participant based on fiber direction images. Both left and right DTI-ALPS indices are calculated as [(Dxx-proj+Dxx-assoc)/(Dyy-proj+Dzz-assoc)] on each side, respectively. Average DTI-ALPS is calculated as the mean of the left and right DTI-ALPS indices. The workflow and illustration for DTI-ALPS are shown in Figure 1.
2. PVS volume: PVS volume was measured based on T1 images as previously described.^15^ Lacunes were first manually identified by an experienced neuroradiologist and manually delineated using ITK-SNAP (http://www.itksnap.org). The signal intensities of PVS tend to be identical to or lower than those of CSF. Therefore, to create PVS maps, we used the already created CSF maps. The manually identified lacunes, the ventricles, and the CSF surrounding the large vessels and outside the brain were removed from the CSF maps to create PVS maps. Each PVS map was manually inspected to ensure that only PVS was included. PVS volumes were calculated in individual subject spaces by summing these binarized corrected maps. Volumes were normalized with respect to total brain volume. Interrater and intrarater reliability metrics were determined by 2 raters using 20 randomly selected scans. The interrater reliability metrics for PVS volumes were SEM 2 mm^3^, mean variability 4.32% (SD, 4.19%), and intraclass correlation coefficient 0.99 across all time points. To obtain the PVS volume in the basal ganglia (BG) and WM regions, we utilized the following steps: first, we applied N4BiasFieldCorrection for preprocessing the T1 images. Subsequently, BG and WM masks were obtained using Freesurfer 7.0 SynthSeg. Finally, the whole-brain PVS mask, BG mask, and WM mask were overlaid using fslstats-V to calculate the PVS volume in each region.

### Statistical Analysis

All the statistical analyses were performed in R-studio (R version 4.1.3). PVS and WMH volume in this study were all normalized by whole brain volume (WM volume+gray matter volume) and log transformed. A false discovery rate correction was used to account for multiple comparisons.

#### Baseline Association Between PVS Markers and CSVD MRI Markers

Linear regression models were performed to explore the relationship between PVS markers and CSVD MRI markers (WMH volume, number of lacunes, number of microbleeds, PSMD, and median MD). In all models, age, sex, and vascular risk factors were included as covariates.

#### Baseline PVS Markers as Predictor for CSVD Progression

A linear mixed model, implemented using the R packages (lmer4 and lmerTest), was used to analyze associations for continuous variables such as WMH volume and conventional DTI markers. For binary variables (lacunes and microbleeds), logistic regression was used. In all analyses, age, sex, and vascular risk factors were included as covariates.

#### Baseline PVS Markers as Predictors for Cognitive Changes and Dementia

Linear mixed models were used to examine associations between PVS markers and changes in cognition. Cox regression models were used to examine the association between baseline PVS markers and incident dementia. Age, sex, NART-IQ, and vascular risk factors were included as covariates; CSVD markers (WMH volume, lacune number, and microbleed number) or median MD or PSMD were then added. Due to the presence of multicollinearity among the CSVD markers (WMH volume, lacune, and microbleed), PVS markers (DTI-ALPS and PVS volume), PSMD, and median MD, a random forest regression analysis was conducted using the party R package to analyze the association between each cognitive performance measure and the independent variables. Age, sex, NART-IQ, CSVD markers (WMH volume, lacune number, and microbleed number), PVS markers (DTI-ALPS and PVS volume), PSMD, and median MD were entered as independent variables. The dependent variables were changes in cognition over a 5-year period within each domain. Besides, dementia and nondementia-ending events were also set as dependent variables.

#### Longitudinal Change of PVS Markers

To study the change of PVS markers over time, we used linear mixed-effects regression with random effects of intercept and linear slope (with respect to time). For subsequent analysis, the slopes were extracted for each patient. To assess whether PVS marker change is correlated with the progression of CSVD MRI markers, linear regression analysis was used for continuous variables (WMH volume, median MD, and PSMD) with age, sex, vascular risk factors, and baseline PVS markers set as covariates. Logistic regression was used to investigate whether a change in lacune or microbleed count was associated with a change in PVS markers.

To assess whether change in PVS markers over the 3 years predicted dementia conversion, Cox regression models were used with age, sex, NART-IQ, vascular risk factors, and baseline PVS markers included as covariates. In further analysis of visible CSVD markers (WMH volume, lacune number, and microbleed number), median MD or PSMD was added as covariate.

## RESULTS

### Baseline Demographics and Imaging Characteristics

A total of 121 patients were recruited to SCANS study, of whom 120 had DTIs available and were included in the baseline analysis. Ninety-nine patients had MRIs at >1 time point and were included in the longitudinal analysis. Patients’ demographics, CSVD burden on MRI, and PVS markers are shown in Table 1. Comparisons of patients included and not included in the longitudinal study are shown in Table S1. Neuropsychological assessments each year are shown in Table S2.

**Table 1.**
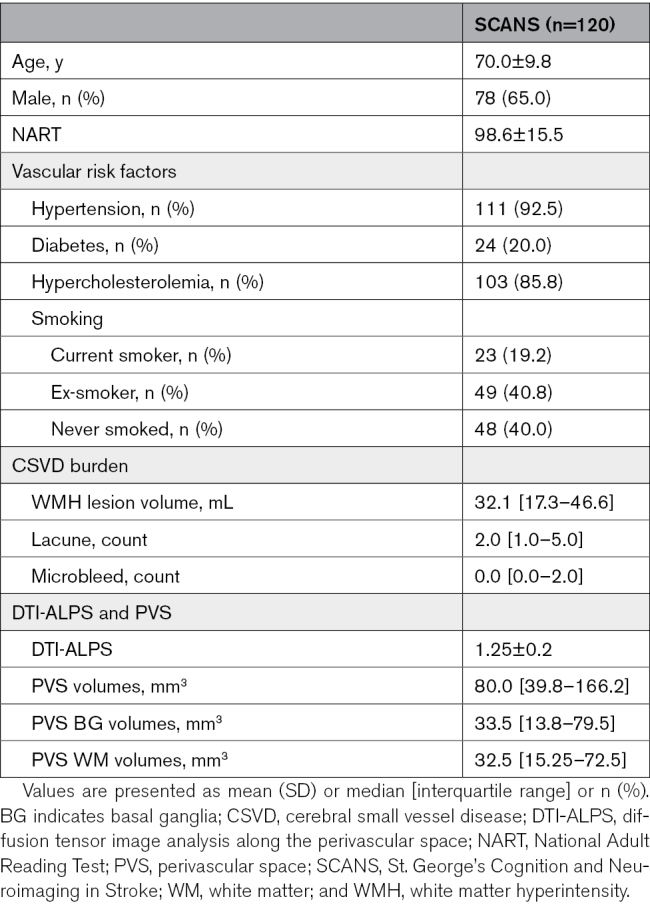
Demographics, CSVD Burden, and PVS Markers in Baseline Participants

### Baseline Associations Between PVS Markers (DTI-ALPS and PVS Volume) and Conventional CSVD Imaging Markers

DTI-ALPS demonstrated significant associations with WMH volume (β=−0.268, *P*=0.005), number of lacunes (β=−0.295, *P*=0.001), PSMD (β=−0.385, *P*<0.001), and median MD (β=−0.392, *P*<0.001) but was not associated with the number of microbleeds (β=−0.110, *P*=0.230; Table 2). In contrast, whole-brain PVS volume was solely associated with the number of lacunes (β=0.292, *P*=0.006). For subregion PVS volume analysis, we did not find significant correlations between BG/WM PVS volume and CSVD imaging makers after multiple comparisons (Table S3).

**Table 2.**
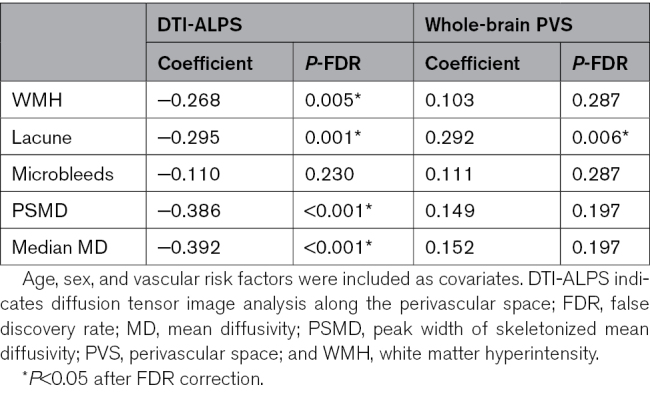
Baseline Associations Between DTI-ALPS and Whole-Brain PVS Volume and Cerebral Small Vessel Disease (CSVD) Markers and Diffusion Tensor Image (DTI) Markers

### Do DTI-ALPS and PVS Volume at Baseline Predict CSVD Progression as Assessed With Conventional MRI Markers?

We next determined whether DTI-ALPS and PVS volume at baseline predicted CSVD progression as assessed on MRI over the 3-year MRI follow-up period using linear mixed models for continuous variables and logistic regressions for binary variables. Results are shown in Table S4. Baseline DTI-ALPS was associated with WMH progression, but this did not survive false discovery rate correction for multiple comparisons. There were no associations with whole-brain PVS volume. For subregion PVS volume analysis, both BG and WM PVS volumes did not show associations with CSVD progression (Table S5).

### Do Baseline DTI-ALPS and PVS Volume Predict Cognitive Decline and Dementia?

We next determined whether markers at baseline predicted cognitive decline and incident dementia over the 5-year follow-up. Results are shown in Table 3. Baseline DTI-ALPS predicted changes in global cognition (β=0.142, *P*=0.032), executive function (β=0.287, *P*=0.027), and long-term memory subdomains (β=0.228, *P*=0.027) but did not predict changes in processing speed (β=0.052, *P*=0.608). Results remained consistent after controlling for conventional DTI (either median MD or PSMD). In contrast, whole-brain PVS volume at baseline did not predict any cognitive changes, and the same was observed for PVS volume in the BG and WM regions (Table S6).

**Table 3.**
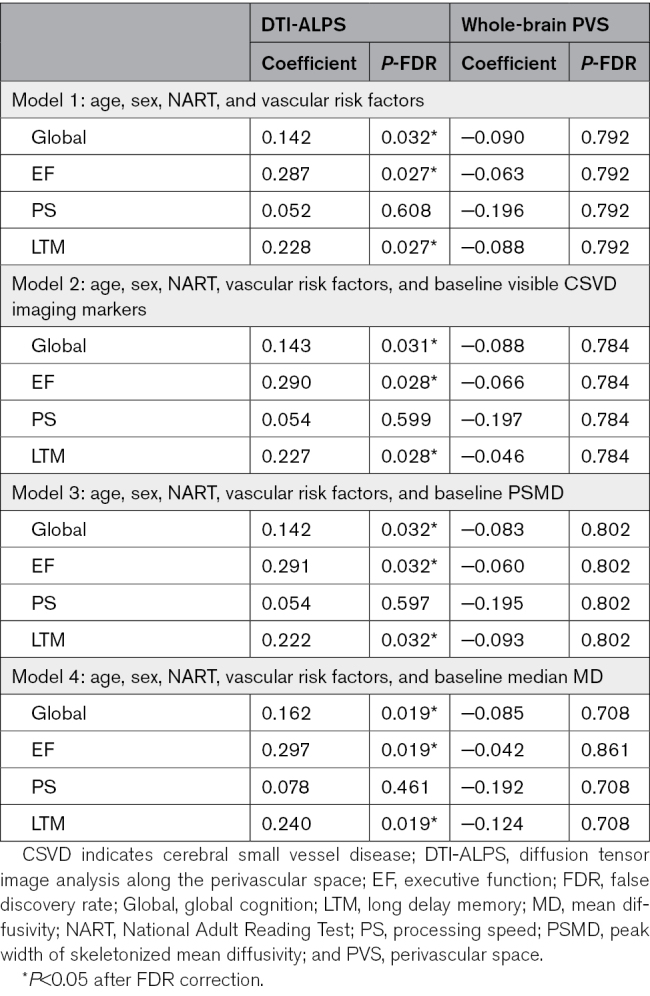
Baseline DTI-ALPS and PVS as Predictors for Cognitive Decline Over 5 Years

**Figure 2.**
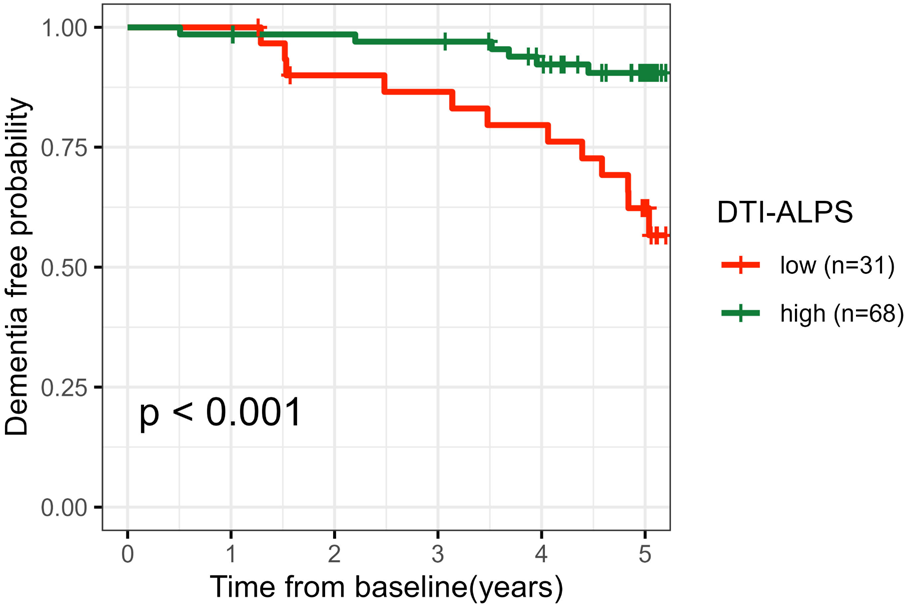
Survival curves according to baseline diffusion tensor image analysis along the perivascular space (DTI-ALPS).

Eighteen participants (18.2%) developed dementia during follow-up. Baseline DTI-ALPS predicted incident dementia (hazard ratio [HR], 0.328; *P*<0.001), with higher baseline DTI-ALPS associated with a lower risk of dementia. The association persisted after controlling for visible CSVD markers (WMH, lacune, and microbleed; HR, 0.316; *P*<0.001), median MD (HR, 0.290; *P*<0.001), and PSMD (HR, 0.327; *P*=0.002). Survival curves were plotted according to the best DTI-ALPS cutoff (1.18; calculated by R package cutpointr), which showed baseline lower DTI-ALPS was associated with a higher risk of dementia (*P*<0.001; Figure 2). In contrast, baseline whole-brain PVS volume did not predict dementia conversion (HR, 0.761; *P*=0.677). Details are shown in Table 4.

**Table 4.**
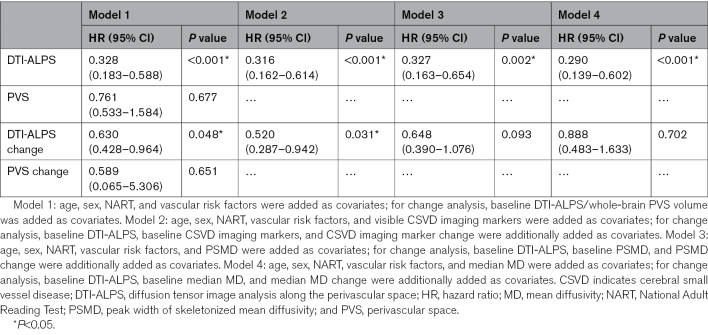
DTI-ALPS and PVS as a Predictor for Incident Dementia During 5-Year Follow-Up

For random forest analysis, we found baseline DTI-ALPS exhibited better performance compared with all other CSVD markers in terms of changes in global cognition and processing speed over the 5-year period. Regarding change in executive function, lacunes explained more variance than DTI-ALPS, although DTI-ALPS also demonstrated relatively high variable importance. However, for long-term memory, PSMD displayed slightly higher variable importance than DTI-ALPS. For further details, see Figure S2. Regarding predicting dementia, DTI-ALPS displayed the highest variable importance compared with all other variables (Figure S3).

### Longitudinal Change of DTI-ALPS and PVS Volume and Their Association With MRI Markers of CSVD Progression and Development of Dementia

During the 3-year MRI follow-up period, there was a significant decline in DTI-ALPS (β=−0.010, *P*<0.001) but no change in whole-brain PVS volume (β=−1.244×10^−3^, *P*=0.677; Figure S4). BG PVS volume (β=8.987×10^−4^, *P*=0.629) and WM PVS volume (β=−1.584×10^−3^, *P*=0.326) also showed no change (Figure S5). There was a marked temporal variation in PVS volume in some individuals over time, with some increasing and others decreasing (Figure S4).

Change in DTI-ALPS was associated with change in median MD (r=−0.314, *P*=0.013), but there was no association with PSMD (r=−0.102, *P*=0.399). There was no correlation between change in DTI-ALPS and progression of WMH (r=−0.104, *P*=0.399), change in the number of lacunes (r=−0.447, *P*=0.399), or microbleeds (r=−0.163, *P*=0.470) after false discovery rate multiple comparisons. Changes in whole-brain PVS, BG PVS, and WM PVS volumes did not correlate with changes in any CSVD marker (Table S7).

Change in DTI-ALPS predicted dementia conversion (HR, 0.630; *P*=0.048), but when PSMD and median MD were entered as covariates, the association was no longer significant. A change in whole-brain PVS volume did not predict dementia conversion. More details are shown in Table 4. Changes in BG PVS and WM PVS volumes did not predict dementia conversion (Table S8).

## DISCUSSION

In this study, we found that a novel magnetic resonance marker of PVS function (DTI-ALPS) predicted both dementia and cognitive decline over a 5-year follow-up period in patients with CSVD. Many previous studies have shown associations between DTI-ALPS and cognition in CSVD, but these have been largely cross-sectional,^20,30,31^ with few longitudinal studies,^21^ and cannot determine whether PVS diffusion, as measured on DTI-ALPS, causally contributes to CSVD progression. Our data, demonstrating its predictive ability for dementia over a 5-year follow-up, support a potential causal role in cognitive impairment in CSVD. We showed that baseline DTI-ALPS not only predicted cognitive decline and incident dementia but also that change in DTI-ALPS metrics over the initial 3-year period, when annual MRI scans were performed, predicted dementia over the 5-year follow-up period, further supporting a causal association.

DTI-ALPS is derived from the DTI sequence and measures diffusion along the PVS. However, it is possible that it might also be influenced by and merely act as another measure of conventional DTI metrics such as MD, which itself has been shown to be a strong predictor of cognitive decline and dementia.^25^ Therefore, we controlled for 2 conventional DTI metrics in our analysis: MD and PSMD, an automated method of measuring diffusivity within the WM tracts. Associations between baseline DTI-ALPS and both cognition and dementia remained significant after controlling for median MD or PSMD. The associations between changes in DTI-ALPS and dementia did not remain after controlling for PSMD or MD. This suggests that part of the DTI-ALPS signal change could be describing conventional DTI metrics such as MD.

DTI-ALPS is being increasingly used to explore the role of glymphatic dysfunction in a variety of neurological diseases. It was first proposed in 2017^17^ and assesses the movement of water molecules in different directions in the PVS. Specifically, it allows evaluation of diffusivity parallel to PVS surrounding medullary veins at the level of the lateral ventricles. A validation study reported a good correlation with glymphatic function determined using classical contrast injection techniques,^18^ and it has good reproducibility both in single-center^32^ and across scanner studies.^33^ Our data showed consistent results across time points with a gradual decline over time. Recent studies have shown DTI-ALPS changes in patients with a variety of neurological diseases, including Alzheimer disease,^17^ Parkinson disease,^34^ and traumatic brain injury.^35^ Many cross-sectional studies have also implicated glymphatic dysfunction assessed by DTI-ALPS in patients with CSVD^18,20^ and vascular risk factors^19^ and have reported associations with the severity of cognitive impairment in both CSVD and vascular cognitive impairment^30,31^ and Alzheimer disease.^36^ However, few studies have determined whether DTI-ALPS changes predict future cognitive decline and dementia. Our study provides novel data supporting this association and is consistent with recent data from Parkinson disease reporting that DTI-ALPS predicted future cognitive decline.^37^ Taken together, our data support a role of glymphatic dysfunction in mediating cognitive decline in CSVD, consistent with animal data suggesting a role of glymphatic dysfunction in CSVD progression. Rodent models of CSVD, particularly spontaneous hypertensive rats, have demonstrated glymphatic dysfunction, including reduced CSF fluid dynamics evaluated by dynamic contrast-enhanced MRI and loss of aquaporin-4, a crucial channel for glymphatic function.^38,39^

We further explored associations between DTI-ALPS and other markers of CSVD burden to determine whether the association was mediated via other markers of CSVD severity. In cross-sectional analysis, DTI-ALPS was highly significantly associated with WMH volume, number of lacunes, and both PSMD and median MD, consistent with previous reports.^20^ However, there was little association between DTI-ALPS and the progression of other neuroimaging markers of CSVD. Baseline DTI-ALPS was only associated with WMH progression, but the association did not survive correction for multiple comparisons. A change in DTI-ALPS was not associated with either the progression of WMH or a change in the number of lacunes. It was associated with a change in median MD, but caution needs to be made in interpreting this due to DTI-ALPS being derived from the DTI sequence. We cannot exclude an association with WMH, and the 3-year MRI follow-up may provide too short a time to detect associations. However, it raises the possibility that the association between DTI-ALPS and cognitive decline may not be mediated by conventional MRI markers of CSVD.

In contrast to associations with DTI-ALPS, we found no associations between baseline PVS, or change in PVS volume, and cognitive decline or dementia. This is consistent with and extends the results, of our previous study.^15^ There have been conflicting results from studies of the associations between PVS and cognition, but a meta-analysis of 5 population-based studies failed to find an association, consistent with our findings.^14^ Several factors could underlie this lack of association. Enlarged PVS may reflect an end stage of glymphatic dysfunction, in contrast to the more dynamic changes detected on DTI-ALPS, which are likely to detect glymphatic dysfunction at an earlier stage. We also found that, while interobserver agreement in measurement of PVS volume was high, PVS volume varied markedly across time points in some individuals, and this would reduce the chance of detecting any association. Furthermore, from a mechanistic perspective, PVS volume primarily assesses the periarteriolar spaces,^40^ which are more closely related to influx, while DTI-ALPS evaluates diffusivity along the deep medullary space,^2^ effectively detecting the efflux ability of glymphatic function and may be more relevant to the retention of toxic substances in the brain.

Our study has several strengths. We used a prospective longitudinal cohort, allowing us to determine whether DTI-ALPS and PVS predict future dementia risk. Importantly, we controlled for conventional DTI markers in our analysis. It also has limitations. Imaging was performed on a 1.5T scanner, and the resolution of the DTI was not as high as on newer scanners. PVS were identified and differentiated from lacunar infarcts by an expert neuroradiologist. The neuroradiologist was blinded to clinical and cognitive information. However, we did not assess the interobserver measurement of PVS volume. The exclusion of older subjects with lower NART-IQ scores might introduce selection bias into our findings, and further studies with older subjects are required to assess this.

In conclusion, we have shown that DTI-ALPS, but not PVS volume, predicts future dementia risk. Our results would support the role of glymphatic dysfunction in cognitive impairment in CSVD, although the weakening of longitudinal associations after controlling for MD suggests that part of the DTI-ALPS signal may represent conventional DTI metrics such as MD. Further studies are required with larger sample sizes, longitudinal follow-up, and the inclusion of more patients with mild CSVD. It is important that these control for conventional DTI markers, as we did in our study. If these replicate an association between DTI-ALPS and the prediction of future cognitive decline and dementia, then DTI-ALPS could potentially serve as a marker for predicting the risk of future dementia.

## ARTICLE INFORMATION

### Sources of Funding

This study was funded by a British Heart Foundation program grant (RG/F/22/110052). Infrastructural support was provided by Cambridge British Heart Foundation Centre of Research Excellence (RE/18/1/34212) and Cambridge University Hospitals National Institute for Health and Care Research Biomedical Research Centre (NIHR203312)

### Disclosures

Dr Tozer was funded by the Medical Research Council. H.S. Markus discloses grant support from the British Heart Foundation. The other author reports no conflict.

### Supplemental Material

Tables S1–S8

Figures S1–S5

## Supplementary Material



## References

[1] Markus HS de Leeuw FE. Cerebral small vessel disease: recent advances and future directions. Int J Stroke. 2023;18:4–14. doi: 10.1177/17474930221144911 36575578 10.1177/17474930221144911 PMC9806465

[2] Sweeney MD Montagne A Sagare AP Nation DA Schneider LS Chui HC Harrington MG Pa J Law M Wang DJ. Vascular dysfunction—the disregarded partner of Alzheimer’s disease. Alzheimer’s Dement. 2019;15:158–167. doi: 10.1016/j.jalz.2018.07.222 30642436 10.1016/j.jalz.2018.07.222 PMC6338083

[3] Brown R Benveniste H Black SE Charpak S Dichgans M Joutel A Nedergaard M Smith KJ Zlokovic BV Wardlaw JM. Understanding the role of the perivascular space in cerebral small vessel disease. Cardiovasc Res. 2018;114:1462–1473. doi: 10.1093/cvr/cvy113 29726891 10.1093/cvr/cvy113 PMC6455920

[4] Wardlaw JM Benveniste H Nedergaard M Zlokovic BV Mestre H Lee H Doubal FN Brown R Ramirez J MacIntosh BJ; Colleagues From the Fondation Leducq Transatlantic Network of Excellence on the Role of the Perivascular Space in Cerebral Small Vessel Disease. Perivascular spaces in the brain: anatomy, physiology and pathology. Nat Rev Neurol. 2020;16:137–153. doi: 10.1038/s41582-020-0312-z 32094487 10.1038/s41582-020-0312-z

[5] Mestre H Kostrikov S Mehta RI Nedergaard M. Perivascular spaces, glymphatic dysfunction, and small vessel disease. Clin Sci (Lond). 2017;131:2257–2274. doi: 10.1042/CS20160381 28798076 10.1042/CS20160381 PMC5567781

[6] Potter GM Doubal FN Jackson CA Chappell FM Sudlow CL Dennis MS Wardlaw JM. Enlarged perivascular spaces and cerebral small vessel disease. Int J Stroke. 2015;10:376–381. doi: 10.1111/ijs.12054 23692610 10.1111/ijs.12054 PMC4463944

[7] Zhu YC Tzourio C Soumaré A Mazoyer B Dufouil C Chabriat H. Severity of dilated Virchow-Robin spaces is associated with age, blood pressure, and MRI markers of small vessel disease: a population-based study. Stroke. 2010;41:2483–2490. doi: 10.1161/STROKEAHA.110.591586 20864661 10.1161/STROKEAHA.110.591586

[8] Wang S Huang P Zhang R Hong H Jiaerken Y Lian C Yu X Luo X Li K Zeng Q. Quantity and morphology of perivascular spaces: associations with vascular risk factors and cerebral small vessel disease. J Magn Reson Imaging. 2021;54:1326–1336. doi: 10.1002/jmri.27702 33998738 10.1002/jmri.27702

[9] Potter GM Chappell FM Morris Z Wardlaw JM. Cerebral perivascular spaces visible on magnetic resonance imaging: development of a qualitative rating scale and its observer reliability. Cerebrovasc Dis. 2015;39:224–231. doi: 10.1159/000375153 25823458 10.1159/000375153 PMC4386144

[10] Huang P Zhang R Jiaerken Y Wang S Yu W Hong H Lian C Li K Zeng Q Luo X. Deep white matter hyperintensity is associated with the dilation of perivascular space. J Cereb Blood Flow Metab. 2021;41:2370–2380. doi: 10.1177/0271678X211002279 33757317 10.1177/0271678X211002279 PMC8393291

[11] Huo Y Wang Y Guo C Liu Q Shan L Liu M Wu H Li G Lv H Lu L. Deep white matter hyperintensity is spatially correlated to MRI-visible perivascular spaces in cerebral small vessel disease on 7 Tesla MRI. Stroke Vasc Neurol. 2023;8:144–150. doi: 10.1136/svn-2022-001611 36170993 10.1136/svn-2022-001611 PMC10176991

[12] Romero JF Pinheiro A Aparicio HJ DeCarli CS Demissie S Seshadri S. MRI-visible perivascular spaces and risk of incident dementia. Neurology. 2022;99:e2561–e2571. doi: 10.1212/WNL.0000000000201293. 36175148 10.1212/WNL.0000000000201293 PMC9754649

[13] Paradise M Crawford JD Lam BC Wen W Kochan NA Makkar S Dawes L Trollor J Draper B Brodaty H. Association of dilated perivascular spaces with cognitive decline and incident dementia. Neurology. 2021;96:e1501–e1511. 33504642 10.1212/WNL.0000000000011537 PMC8032377

[14] Hilal S Tan CS Adams HH Habes M Mok V Venketasubramanian N Hofer E Ikram MK Abrigo J Vernooij MW. Enlarged perivascular spaces and cognition: a meta-analysis of 5 population-based studies. Neurology. 2018;91:e832–e842. 30068634 10.1212/WNL.0000000000006079 PMC6133622

[15] Benjamin P Trippier S Lawrence AJ Lambert C Zeestraten E Williams OA Patel B Morris RG Barrick TR MacKinnon AD. Lacunar infarcts, but not perivascular spaces, are predictors of cognitive decline in cerebral small-vessel disease. Stroke. 2018;49:586–593. doi: 10.1161/STROKEAHA.117.017526 29438074 10.1161/STROKEAHA.117.017526 PMC5832012

[16] Gertje EC van Westen D Panizo C Mattsson-Carlgren N Hansson O. Association of enlarged perivascular spaces and measures of small vessel and Alzheimer disease. Neurology. 2021;96:e193–e202. doi: 10.1212/WNL.0000000000011046 33046608 10.1212/WNL.0000000000011046

[17] Taoka T Masutani Y Kawai H Nakane T Matsuoka K Yasuno F Kishimoto T Naganawa S. Evaluation of glymphatic system activity with the diffusion MR technique: diffusion tensor image analysis along the perivascular space (DTI-ALPS) in Alzheimer’s disease cases. Jpn J Radiol. 2017;35:172–178. doi: 10.1007/s11604-017-0617-z 28197821 10.1007/s11604-017-0617-z

[18] Zhang W Zhou Y Wang J Gong X Chen Z Zhang X Cai J Chen S Fang L Sun J. Glymphatic clearance function in patients with cerebral small vessel disease. Neuroimage. 2021;238:118257. doi: 10.1016/j.neuroimage.2021.118257 34118396 10.1016/j.neuroimage.2021.118257

[19] Zhang Y Zhang R Ye Y Wang S Jiaerken Y Hong H Li K Zeng Q Luo X Xu X. The influence of demographics and vascular risk factors on glymphatic function measured by diffusion along perivascular space. Front Aging Neurosci. 2021;13:693787. doi: 10.3389/fnagi.2021.693787 34349635 10.3389/fnagi.2021.693787 PMC8328397

[20] Tian Y Cai X Zhou Y Jin A Wang S Yang Y Mei L Jing J Li S Meng X. Impaired glymphatic system as evidenced by low diffusivity along perivascular spaces is associated with cerebral small vessel disease: a population-based study. Stroke Vasc Neurol. 2023;8:svn-2022-002191. 10.1136/svn-2022-002191 PMC10647865 37045543

[21] Wang J Zhou Y Zhang K Ran W Zhu X Zhong W Chen Y Li J Sun J Lou M. Glymphatic function plays a protective role in ageing-related cognitive decline. Age Ageing. 2023;52:afad107. doi: 10.1093/ageing/afad107 37392401 10.1093/ageing/afad107 PMC10314787

[22] Zeestraten EA Lawrence AJ Lambert C Benjamin P Brookes RL Mackinnon AD Morris RG Barrick TR Markus HS. Change in multimodal MRI markers predicts dementia risk in cerebral small vessel disease. Neurology. 2017;89:1869–1876. doi: 10.1212/WNL.0000000000004594 28978655 10.1212/WNL.0000000000004594 PMC5664300

[23] von Elm E Altman DG Egger M Pocock SJ Gøtzsche PC Vandenbroucke JP; STROBE Initiative. The Strengthening the Reporting of Observational Studies in Epidemiology (STROBE) statement: guidelines for reporting observational studies. Lancet. 2007;370:1453–1457. doi: 10.1016/S0140-6736(07)61602-X 18064739 10.1016/S0140-6736(07)61602-X

[24] Lawrence AJ Patel B Morris RG MacKinnon AD Rich PM Barrick TR Markus HS. Mechanisms of cognitive impairment in cerebral small vessel disease: multimodal MRI results from the St George’s cognition and neuroimaging in stroke (SCANS) study. PLoS One. 2013;8:e61014. doi: 10.1371/journal.pone.0061014 23613774 10.1371/journal.pone.0061014 PMC3632543

[25] Egle M Hilal S Tuladhar AM Pirpamer L Hofer E Duering M Wason J Morris RG Dichgans M Schmidt R. Prediction of dementia using diffusion tensor MRI measures: the OPTIMAL collaboration. J Neurol Neurosurg Psychiatry. 2022;93:14–23. doi: 10.1136/jnnp-2021-326571 34509999 10.1136/jnnp-2021-326571

[26] Lawrence AJ Zeestraten EA Benjamin P Lambert CP Morris RG Barrick TR Markus HS. Longitudinal decline in structural networks predicts dementia in cerebral small vessel disease. Neurology. 2018;90:e1898–e1910. doi: 10.1212/WNL.0000000000005551 29695593 10.1212/WNL.0000000000005551 PMC5962914

[27] Baykara E Gesierich B Adam R Tuladhar AM Biesbroek JM Koek HL Ropele S Jouvent E Chabriat H Ertl-Wagner B; Alzheimer’s Disease Neuroimaging Initiative. A novel imaging marker for small vessel disease based on skeletonization of white matter tracts and diffusion histograms. Ann Neurol. 2016;80:581–592. doi: 10.1002/ana.24758 27518166 10.1002/ana.24758

[28] Lambert C Narean JS Benjamin P Zeestraten E Barrick TR Markus HS. Characterising the grey matter correlates of leukoaraiosis in cerebral small vessel disease. NeuroImage Clin. 2015;9:194–205. doi: 10.1016/j.nicl.2015.07.002 26448913 10.1016/j.nicl.2015.07.002 PMC4564392

[29] Lawrence AJ Chung AW Morris RG Markus HS Barrick TR. Structural network efficiency is associated with cognitive impairment in small-vessel disease. Neurology. 2014;83:304–311. doi: 10.1212/WNL.0000000000000612 24951477 10.1212/WNL.0000000000000612 PMC4115608

[30] Song H Ruan Z Gao L Lv D Sun D Li Z Zhang R Zhou X Xu H Zhang J. Structural network efficiency mediates the association between glymphatic function and cognition in mild VCI: a DTI-ALPS study. Front Aging Neurosci. 2022;14:974114. doi: 10.3389/fnagi.2022.974114 36466598 10.3389/fnagi.2022.974114 PMC9708722

[31] Ke Z Mo Y Li J Yang D Huang L Yang Z Qin R Mao C Lv W Huang Y. Glymphatic dysfunction mediates the influence of white matter hyperintensities on episodic memory in cerebral small vessel disease. Brain Sci. 2022;12:1611. doi: 10.3390/brainsci12121611 36552071 10.3390/brainsci12121611 PMC9775074

[32] Taoka T Ito R Nakamichi R Kamagata K Sakai M Kawai H Nakane T Abe T Ichikawa K Kikuta J. Reproducibility of diffusion tensor image analysis along the perivascular space (DTI-ALPS) for evaluating interstitial fluid diffusivity and glymphatic function: Changes in Alps index on Multiple Condition Acquisition Experiment (CHAMONIX) study. Jpn J Radiol. 2022;40:147–158. doi: 10.1007/s11604-021-01187-5 34390452 10.1007/s11604-021-01187-5 PMC8803717

[33] Liu X Barisano G Shao X Jann K Ringman JM Lu H Arfanakis K Caprihan A DeCarli C Gold BT. Cross-vendor test-retest validation of diffusion tensor image analysis along the perivascular space (DTI-ALPS) for evaluating glymphatic system function [published online May 26, 2023]. Aging Dis. 2023. doi: 10.14336/AD.2023.0321-2 10.14336/AD.2023.0321-2 PMC11272201 37307817

[34] Si X Guo T Wang Z Fang Y Gu L Cao L Yang W Gao T Song Z Tian J. Neuroimaging evidence of glymphatic system dysfunction in possible REM sleep behavior disorder and Parkinson’s disease. NPJ Parkinson’s Dis. 2022;8:54. doi: 10.1038/s41531-022-00316-9 35487930 10.1038/s41531-022-00316-9 PMC9055043

[35] Butler T Zhou L Ozsahin I Wang XH Garetti J Zetterberg H Blennow K Jamison K de Leon MJ Li Y. Glymphatic clearance estimated using diffusion tensor imaging along perivascular spaces is reduced after traumatic brain injury and correlates with plasma neurofilament light, a biomarker of injury severity. Brain Commun. 2023;5:fcad134. doi: 10.1093/braincomms/fcad134 37188222 10.1093/braincomms/fcad134 PMC10176239

[36] Hsu JL Wei YC Toh CH Hsiao IT Lin KJ Yen TC Liao MF Ro LS. Magnetic resonance images implicate that glymphatic alterations mediate cognitive dysfunction in Alzheimer disease. Ann Neurol. 2023;93:164–174. doi: 10.1002/ana.26516 36214568 10.1002/ana.26516 PMC10091747

[37] He P Shi L Li Y Duan Q Qiu Y Feng S Gao Y Luo Y Ma G Zhang Y. The association of the glymphatic function with Parkinson’s disease symptoms: neuroimaging evidence from longitudinal and cross-sectional studies. Ann Neurol. 2023;94:672–683. doi: 10.1002/ana.26729 37377170 10.1002/ana.26729

[38] Mortensen KN Sanggaard S Mestre H Lee H Kostrikov S Xavier AL Gjedde A Benveniste H Nedergaard M. Impaired glymphatic transport in spontaneously hypertensive rats. J Neurosci. 2019;39:6365–6377. doi: 10.1523/jneurosci.1974-18.2019 31209176 10.1523/JNEUROSCI.1974-18.2019 PMC6687896

[39] Naessens DM Coolen BF de Vos J VanBavel E Strijkers GJ Bakker EN. Altered brain fluid management in a rat model of arterial hypertension. Fluids Barriers CNS. 2020;17:1–13. 32590994 10.1186/s12987-020-00203-6 PMC7318739

[40] Bouvy WH Biessels GJ Kuijf HJ Kappelle LJ Luijten PR Zwanenburg JJ. Visualization of perivascular spaces and perforating arteries with 7 T magnetic resonance imaging. Invest Radiol. 2014;49:307–313. doi: 10.1097/RLI.0000000000000027 24473365 10.1097/RLI.0000000000000027

