# Supplementary material for: Relationship of Perivascular Space Markers With Incident Dementia in Cerebral Small Vessel Disease
Source: Stroke. 2024 Mar 11;55(4):1032–40. doi: 10.1161/STROKEAHA.123.045857 (PMC10962441; doi:10.1161/STROKEAHA.123.045857)
Supplement: Supplementary file 2 [file str-55-1032-s002.pdf]

SUPPLEMENTAL MATERIAL

Table S1 Demographics, CSVD burden and PVS markers comparison between participants included and not included in following analysis

	Included (n =99)	Not included (n =21)	p-value
Age, years	68.9±10.0	75.2±6.9	0.001*
Male, n(%)	66(66.7%)	12(57.14%)	0.562
NART	100.1±15.3	91.9±15.0	0.032*
**Vascular Risk factors**			
Hypertension, n (%)	92(92.9%)	19(90.5%)	1.000
Diabetes, n (%)	19(19.2%)	5(23.8%)	0.857
Hypercholesterolemia, n (%)	85(85.9%)	18(85.7%)	1.000
Smoking			0.443
current smoker, n (%)	21(21.2%)	2(9.5%)	
ex-smoker, n (%)	40(40.4%)	9(42.9%)	
never smoking, n (%)	38(38.4%)	10(47.6%)	
**CSVD burden**			
WMH, ml	31.9[17.5-46.8]	32.4[16.1-46.5]	0.966
Lacune, count	2.0 [1.0-5.0]	3.0 [1.0-5.0]	0.834
Microbleed, count	0.0[0.0-2.0]	0.0[0.0-1.0]	0.600
**DTI-ALPS and PVS**			
DTI-ALPS	1.25 ± 0.1	1.27 ± 0.2	0.577
Whole brain PVS volumes, mm^3	82.0[39.5-166.5]	66.0[40.0-160.0]	0.664
BG PVS volume, mm^3	34.0[13.5-82.5]	33.0[17.0-44.0]	0.463
WM PVS volume, mm^3	32.0[16.0-70.5]	37.0[12.0-81.0]	0.757

Values are presented as mean (SD) or median [IQR] or n (%). NART = National Adult Reading Test; CSVD= cerebral small vessel disease; WMH = white matter hyperintensity; PVS = perivascular space. BG = basal ganglia; WM = white matter; * $p < 0.05$

Table S2. Neuropsychological assessment each year

	Baseline	1-year	2-year	3-year	4-year	5-year
**Global** (mean±SD, n)	-0.568±0.822 (n= 99)	-0.547±0.816 (n= 93)	-0.475±0.873 (n= 76)	-0.357±0.786 (n= 69)	-0.489±0.884 (n= 35)	-0.523±0.901 (n= 53)
**EF** (mean±SD, n)	-0.750±1.057 (n= 99)	-0.866±1.102 (n= 95)	-0.787±1.122 (n= 75)	-0.585±1.029 (n= 69)	-0.677±1.155 (n= 36)	-0.832±1.144 (n= 54)
**PS** (mean±SD, n)	-0.909±0.863 (n= 99)	-0.934±0.853 (n= 92)	-0.867±0.879 (n= 76)	-0.879±0.801 (n= 70)	-1.014±0.862 (n= 33)	-0.973±0.956 (n= 50)
**LTM** (mean±SD, n)	0.032±0.984 (n= 99)	0.182±1.041 (n= 95)	0.307±1.087 (n= 77)	0.527±1.038 (n= 70)	0.266±1.149 (n= 36)	0.304±1.110 (n= 54)

Global = Global cognition; LTM = long delay memory; PS = processing speed; EF = executive function.

Table S3. Baseline associations between **BG-PVS and WM-PVS volume** and conventional CSVD imaging markers

	**BG PVS**		**WM PVS**	
	Coef.	p-FDR	Coef.	p-FDR
WMH	0.089	0.464	0.029	0.759
Lacune	0.231	0.054	0.198	0.135
Microbleed	0.027	0.778	0.081	0.481
PSMD	0.121	0.464	0.111	0.395
Median MD	0.088	0.464	0.139	0.368

WMH = white matter hyperintensity; PSMD = peak width of skeletonized mean diffusivity; MD = mean diffusivity, PVS = perivascular space; BG = basal ganglia; WM = white matter; FDR = False Discovery Rate; *p < 0.05 after FDR correction.

Table S4. Association between baseline DTI-ALPS and PVS volume with CSVD progression

	**DTI-ALPS**		**Whole brain PVS**	
	Coef.	p-FDR	Coef.	p-FDR
WMH	-1.085	0.068	0.043	0.937
Lacune	-2.363	0.196	2.423	0.316
Microbleed	-3.430	0.076	0.090	0.937
PSMD	-0.226	0.118	0.052	0.937
Median MD	-0.120	0.225	0.049	0.937

WMH=white matter hyperintensity; PSMD=peak width of skeletonized mean diffusivity; MD=mean diffusivity, PVS=perivascular space.

Table S5. The association between baseline **BG PVS and WM PVS volume** with CSVD progression

	**BG PVS**		**WM PVS**	
	Coef.	p-FDR	Coef.	p-FDR
WMH	0.671	0.941	-0.973	0.839
Lacune	15.214	0.092	4.889	0.839
Microbleed	0.425	0.941	-1.516	0.839
PSMD	0.423	0.941	-0.116	0.839
Median MD	-0.054	0.941	0.429	0.839

WMH = white matter hyperintensity; PSMD = peak width of skeletonized mean diffusivity; MD = mean diffusivity, PVS = perivascular space; BG = basal ganglia; WM = white matter; FDR = False Discovery Rate; *$p < 0.05$ after FDR correction.

Table S6. Baseline **BG PVS and WM PVS volume** as predictors for cognitive decline over 5 years

	**BG-PVS**		**WM-PVS**	
	Coef.	p-FDR	Coef.	p-FDR
Model 1: Age, sex, NART, and vascular risk factors				
Global	-0.200	0.666	0.006	0.982
EF	-0.196	0.694	0.333	0.828
PS	-0.355	0.666	-0.337	0.828
LTM	-0.121	0.694	-0.187	0.828
Model 2: Age, sex, NART, vascular risk factors, and **baseline visible CSVD imaging markers**				
Global	-0.202	0.656	0.019	0.941
EF	-0.207	0.670	0.333	0.911
PS	-0.357	0.656	-0.336	0.911
LTM	-0.130	0.670	-0.091	0.911
Model 2: Age, sex, NART, vascular risk factors, and **baseline PSMD**				
Global	-0.201	0.582	0.032	0.900
EF	-0.210	0.582	0.344	0.807
PS	-0.333	0.582	-0.338	0.807
LTM	-0.168	0.582	-0.194	0.807
Model 3: Age, sex, NART, vascular risk factors, and **baseline median MD**				
Global	-0.199	0.653	0.025	0.925
EF	-0.196	0.653	0.414	0.682
PS	-0.352	0.653	-0.351	0.682
LTM	-0.136	0.653	-0.248	0.682

MD = mean diffusivity, WMH = white matter hyperintensity; NART = National Adult Reading Test; PVS = perivascular space; CSVD = cerebral small vessel disease; Global = Global cognition; LTM = long delay memory; PS = processing speed; EF = executive function; BG = basal ganglia; WM = white matter; FDR = False Discovery Rate; *p < 0.05 after FDR correction.

Table S7. The association between first 3 years **PVS markers** change and CSVD progression

	DTI-ALPS change		Whole brain PVS change		BG PVS change		WM PVS change	
	Coef.	p-FDR	Coef.	p-FDR	Coef.	p-FDR	Coef.	p-FDR
WMH change	-0.136	0.276	0.400	0.575	0.213	0.850	0.343	0.608
PSMD change	-0.125	0.276	0.423	0.575	0.184	0.850	0.221	0.608
Median MD change	-0.309	0.023*	-0.414	0.575	0.126	0.850	0.345	0.608
Lacune change	-0.595	0.126	-0.376	0.774	-0.024	0.966	-1.590	0.608
Microbleed change	-0.271	0.276	-0.260	0.774	0.218	0.850	-0.467	0.608

PVS = perivascular space; WMH = white matter hyperintensity; PSMD = peak width skeleton mean diffusivity; MD = mean diffusivity. BG = basal ganglia; WM = white matter; FDR = False Discovery Rate; * p-FDR <0.05 after FDR corrected

Table S8. **BG PVS and WM PVS volume** as a predictor for incident dementia during 5-year follow-up

	HR (CI)	p-value
BG PVS	0.754(0.394-1.441)	0.392
WM PVS	1.129(0.697-1.829)	0.622
BG PVS change	1.342(0.229-7.857)	0.744
WM PVS change	0.650(0.126-3.340)	0.606

Age, sex and NART were added as covariates; for change analysis baseline BG-PVS/ WM PVS volume was additionally added as covariates.

*$p < 0.05$. HR = hazard ratio; CI = confidence intervals; PVS = perivascular space; BG = basal ganglia; WM = white matter.

Figure S1. Longitudinal cohort flow chart

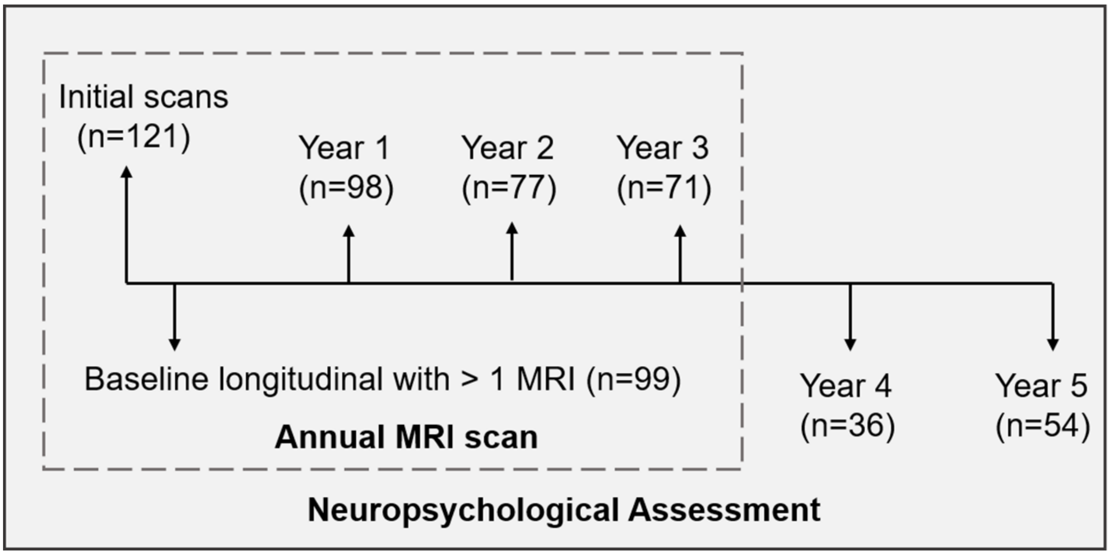

Figure S2. Random forest regression analyses for estimating the relative variable importance of age, sex, NART-IQ, CSVD imaging markers (WMH, lacune, and microbleed), conventional DTI markers (median MD and PSMD), and PVS markers (DTI-ALPS, PVS volume) regarding to cognitive performance changes

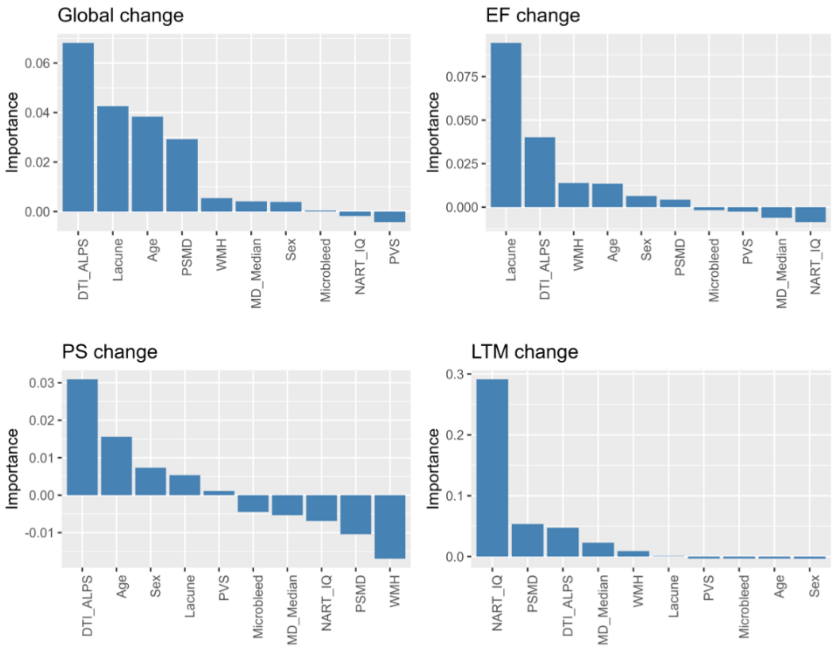

MD = mean diffusivity, WMH = white matter hyperintensity; NART = National Adult Reading Test; PVS = perivascular space; Global = Global cognition; LTM = long delay memory; PS = processing speed; EF = executive function

Figure S3. Random Forest regression analyses for estimating the relative variable importance of age, sex, NART-IQ, CSVD imaging markers (WMH, lacune, and microbleed), conventional DTI markers (median MD and PSMD), and PVS markers (DTI-ALPS, PVS volume) regarding dementia

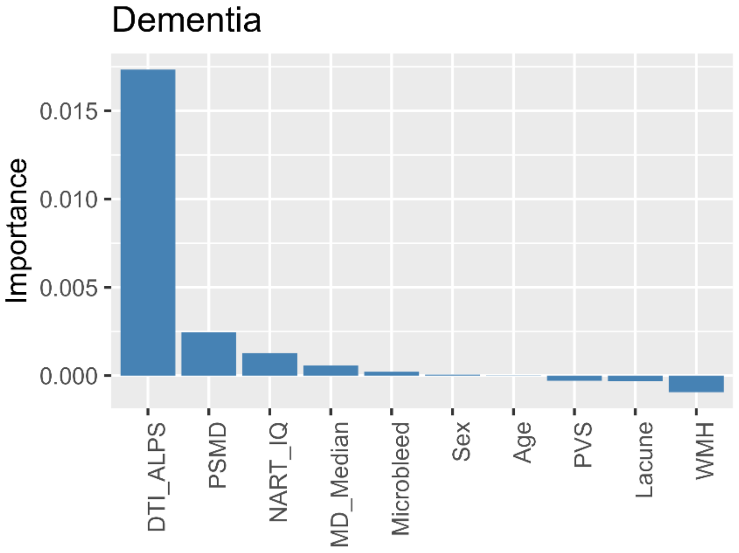

MD = mean diffusivity, WMH = white matter hyperintensity; NART = National Adult Reading Test; PVS = perivascular space; Global = Global cognition; LTM = long delay memory; PS = processing speed; EF = executive function

Figure S4. Longitudinal changes of DTI-ALPS and whole brain PVS volume over 3 years.

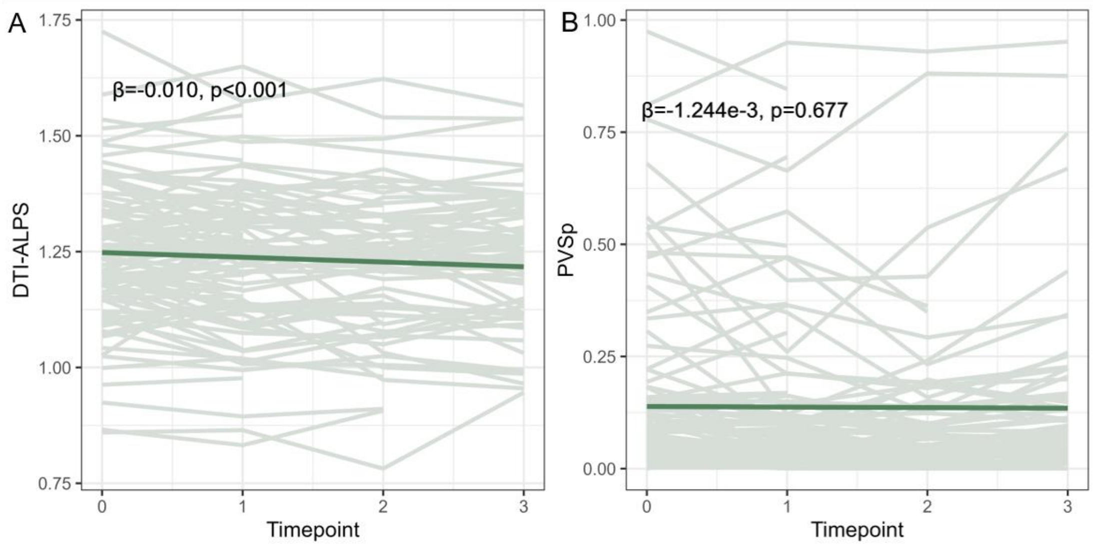

Figure3A: longitudinal changes of DTI-ALPS over 3 years

Figure3B: longitudinal changes of whole brain PVS volume over 3 years, PVSp was calculated by whole brain PVS volume mm^3/brain volume ml

Figure S5. Longitudinal changes of **BG PVS and WM PVS volume** over 3 years

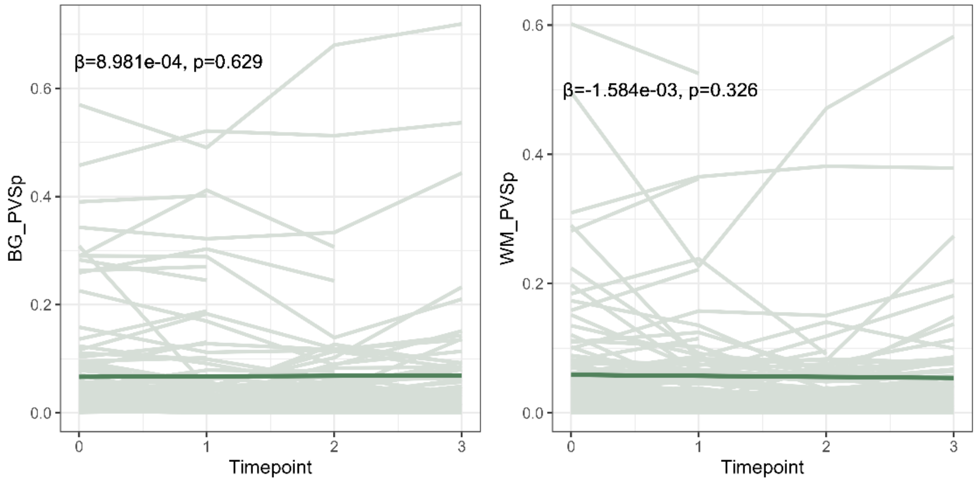

BG_PVSp was calculated by basal ganglia PVS volume mm^3/brain volume ml; WM_PVSp was calculated by white matter PVS volume mm^3/brain volume ml
